# The kleineWeltentdecker App - A smartphone-based developmental diary

**DOI:** 10.3758/s13428-021-01755-7

**Published:** 2022-02-10

**Authors:** Moritz M. Daum, Marco Bleiker, Stephanie Wermelinger, Ira Kurthen, Laura Maffongelli, Katharina Antognini, Miriam Beisert, Anja Gampe

**Affiliations:** 1 Department of Psychology and Jacobs Center for Productive Youth Development, Developmental Psychology: Infancy and Childhood, University of Zurich, Binzmuehlestrasse 14, Box 21, CH-8050 Zurich, Switzerland; 2 Johannes Gutenberg University Mainz, Mainz, Germany; 3 University of Applied Sciences in Special Needs Education, Zurich, Switzerland; 4 University of Duisburg-Essen, Duisburg, Germany

**Keywords:** Ambulatory assessment, Experience sampling, Longitudinal research design, Smartphone application

## Abstract

Today, a vast number of tools exist to measure development in early childhood in a variety of domains such as cognition, language, or motor, cognition. These tools vary in different aspects. Either children are examined by a trained experimenter, or caregivers fill out questionnaires. The tools are applied in the controlled setting of a laboratory or in the children’s natural environment. While these tools provide a detailed picture of the current state of children’s development, they are at the same time subject to several constraints. Furthermore, the measurement of an individual child’s change of different skills over time requires not only one measurement but high-density longitudinal assessments. These assessments are time-consuming, and the breadth of developmental domains assessed remains limited. In this paper, we present a novel tool to assess the development of skills in different domains, a smartphone-based developmental diary app (the *kleineWeltentdecker App*, henceforth referred to as the *APP* (The German expression “kleine Weltentdecker” can be translated as “young world explorers”.)). By using the APP, caregivers can track changes in their children’s skills during development. Here, we report the construction and validation of the questionnaires embedded in the APP as well as the technical details. Empirical validations with children of different age groups confirmed the robustness of the different measures implemented in the APP. In addition, we report preliminary findings, for example, on children’s communicative development by using existing APP data. This substantiates the validity of the assessment. With the APP, we put a portable tool for the longitudinal documentation of individual children’s development in every caregiver’s pocket, worldwide.

## Challenges in the longitudinal assessment of development

The measurement of developmental change is challenging. Our current knowledge about children’s development results to a large extent from cross-sectional studies. Mostly, different individuals of different ages are tested within a narrow time window. This approach is vital for the assessment of age differences, but it only provides a static picture of current developmental states. As a result, a large amount of research in developmental psychology is dedicated to the description of children’s behavior at different ages. It has therefore become somewhat conventional to describe the earliest manifestations of particular abilities (Adolph et al., 2008). However, already Vygotsky raised the concern that a cross-sectional approach primarily focuses on age-dependent and stable endpoints in development (Vygotsky, 1978). Similarly, Adolph and colleagues stated that this kind of research has resulted in “a gallery of before and after snapshots, studio portraits of newborns, and fossilized milestones” (Adolph et al., 2008, p. 527). With these static developmental pictures, little can be learned about developmental processes.

To a certain degree, this shortcoming is compensated for by longitudinal research paradigms. Here, the same individuals are tested multiple times at predefined measurement points, for example, every month or every year. This approach provides information about individual developmental trajectories by relating early and later developing skills. However, this assumed “gold standard” approach has likewise disadvantages. First, it remains unknown what happens between the different measurement points. According to Adolph and colleagues, “sampling rates typically used by developmental researchers may be inadequate to accurately depict patterns of variability and the shape of developmental change” (Adolph et al., 2008, p. 527). That is, when the sampling rate chosen is too low, it does not allow to identify whether a developmental trajectory reflects a smooth and monotonic improvement, a non-linear trend, or an accelerating or decelerating transformation. Second, even on a small scale, longitudinal studies are often highly resource-intensive. They require an extensive amount of human and financial resources, and often a substantial amount of time. Third, the measurement points are usually determined based on the mean age at which certain developmental milestones are expected to be reached. This limits the validity of standard longitudinal research paradigms because the assumed mean age and the accordingly determined measurement point do not necessarily reflect a single individual’s development (Hamaker, 2012). In addition, the actual moment in which a developmental change occurs is not captured with predefined measurement points.

To overcome this limitation, we present a new smartphone-based developmental diary approach that adapts the Age-of-Attainment (AoA) method (e.g., Eaton et al., 2014). The AoA method has its roots in event-centered approaches (e.g., Campbell & Weech, 1941; Wohlwill, 1973). It does not measure developmental processes by the presence or absence of a developmental milestone, for example, whether or not a 12-month-old already walks independently. Rather, it helps to identify the point in time of the emergence of the skill. This allows researchers to capture the individual age differences at which children reach a specific developmental milestone. As a result, the AoA method helps to shift age from being a predictor of other variables to being the outcome explained by those other variables (Wohlwill, 1973). The age at which children first reach a specific developmental milestone (e.g., independent walking) shows substantial inter-individual variability. Capturing this variability may reveal information about underlying developmental processes, for instance by informing about how skills acquired early relate to later ones (Bornstein et al., 2013; Dinehart & Manfra, 2013). Determining the AoA requires behavioral observation that is of higher frequency than the usual applied yearly or monthly observations (optimally, a 24/7 tracking of a child’s development). For feasibility reasons, this requires outsourcing data collection from the controlled environment of a laboratory to the home environment of the children and their caregivers. Current technological developments, such as the widespread availability of smartphones, have the potential to overcome the limitations developmental research was facing so far and to facilitate the collection of comprehensive AoA data.

## Skills do not develop independently of each other

In the past, various researchers have described how to implement designs with multiple outcome measures (LoBue et al., 2020; LoBue & Adolph, 2019; Aslin, 2007; Morris et al., 2006). With the present APP, we aim to expand this view by focusing on the second methodological and theoretical challenge of developmental research: Skills do not develop in isolation. Neither do they develop independently from each other nor independently of the environmental context, which also changes at the micro, meso, and macro levels (Bronfenbrenner, 1992). On the contrary, when a particular skill in one domain occurs or changes, skills in other domains often do not remain unaffected (e.g., Smith & Thelen, 2003).

Let’s exemplify this with the development of basic motor skills: Motor development results from the co-occurrence and interactions of basic maturation processes such as the increased myelinization of the cortical-spinal tract (McGraw, 1943; Zelazo, 1998), other physiological systems (muscle strength and the ability to balance Spencer et al., 2000; Adolph et al., 2003), cognitive and perceptual skills, social-emotional change (e.g., the motivation to move independently), experience (adequate opportunities to practice the emerging skill), which are often influenced by cultural and historical differences in child-rearing practices (Adolph & Hoch, 2019). Vice versa, the development of motor skills is strongly influenced and refined by perceptual, cognitive, motivational skills as well as by cultural and historical differences in child-rearing practices (Adolph & Hoch, 2019). And vice versag the acquisition of new motor skills lays the cornerstone for the emergence and refinement of skills in other domains (Soska et al.,, 2015; for overviews, see Campos et al.,, 2000 and Gredebäck et al.,, 2021). For example, changes in locomotion result in changes in perception: Crawling infants’ look down at the floor to a great extent. In contrast, walking infants direct their gaze at their caregivers and objects in the environment (Kretch et al., 2014). Furthermore, locomotion influences infants’ cognitive skills (Campos et al., 2000), such as their mental rotation of objects: Crawling infants show better mental rotation than non-crawling infants (Schwarzer et al., 2013). Mental rotation is further positively influenced by the infants’ general motor experience (Frick & Wang, 2013; Möhring & Frick, 2013). Also, fine-motor skills (Dinehart & Manfra, 2013) and early action experiences (Bornstein et al., 2013) are significantly related to later academic achievement. Concerning the cultural and historical context, it has been shown that the position in which children sleep (supine or prone) has an impact on the age of acquisition of several motor milestones. Compared to supine sleepers, prone sleepers start earlier rolling prone to supine, tripod sitting, creeping, crawling, and pulling to stand (Davis et al., 1998). The American Academy of Pediatrics recommended in 1992 that infants should be placed on their side or back for sleep (Pediatrics, 1992) to reduce the incidence of sudden infant death syndrome. With this intervention, the percentage of infants sleeping prone has decreased and, accordingly, the age when different motor skills are acquired has increased. This shows how the context in which children grow up provides different opportunities resulting in different developmental trajectories.

While knowledge about specific interrelations such as the ones just reported is increasing, the assessment of the development of the interrelations between skills in different domains and, in particular, their dynamic interaction over time remains limited. The developmental diary approach presented here implements the following features: It includes the development of skills in different domains (cognition, language, motor, and social and emotional skills). The temporal assessment is shorter compared to a large number of longitudinal studies. It relates the development of these different domains to each other. Finally, it considers contextual factors such as the language(s) spoken by the child and the caregivers, and the caregivers’ cultural, educational, and economic background.

## Goals of the kleineWeltentdecker App (APP)

To address these challenges, we developed the kleineWeltentdecker App (henceforth referred to as the APP), a smartphone-based digital developmental diary application. With the APP, we provide a tool to the caregivers to document the development of their children from age 0 to 6. At the same time, caregivers share the data of their children’s development anonymously with our research unit (see also “4”). With this participatory science approach (“caregiver-as-a-researcher”), the APP allows acquiring and analyzing longitudinal data at a relatively high temporal resolution optimally at the exact moment when a developmental change occurs. The following three goals drove the development of this research tool and its related research.

### Goal 1: Establish a comprehensive data set of child development from age 0 to 6, within and across individuals

Given the ubiquity of smartphones worldwide, the range of use of the APP is not limited to specific regions or countries. This aspect facilitates the analysis of the variability of behavior and its development regarding contextual aspects such as culture, SES, language background, and many other demographic and family factors. The acquired data are therefore subject to analyses for the following major purposes: 1) It allows an in-depth analysis of the individual developmental trajectories of major developmental domains. 2) It allows analysis of the dynamically changing interrelations and inter-dependencies of the development in the individual domains. With this approach, developmental trajectories can be compared within and between individuals, or within and between cultures, which helps to identify developmental specificities and universals.

### Goal 2: Account for the variability on development across cultures

One issue of increasing importance in developmental science is the variability in children’s development. Previous research on psychology in general and in developmental psychology in particular is based on data from WEIRD (Western, Educated, Industrialized, Rich, and Democratic) populations (e.g., Nielsen et al., 2017). There is a growing number or researchers who argue that this approach undermines the variability of behavior and development across the globe. Henrich et al., (2010b) state that their “findings suggest that members of WEIRD societies, including young children, are among the least representative populations one could find for generalising about humans” (p. 61). A bias towards WEIRD samples may result in that findings, which are specific to a particular culture, are falsely being interpreted as universal traits (Henrich et al., 2010a; Nielsen et al., 2017). Accordingly, the second goal of the APP is to provide a tool that is not (or at least much less) restricted to the collection of data within a narrow range of participants but is - optimally - available worldwide. In a first step, we implemented the APP in four different languages: British English, French, German, and Italian.

We are aware that, currently, the APP asks caregivers about their children’s skills in a fixed and to some extent “WEIRD”-based order and children from different cultures may develop in a different order. However, the current approach will help to identify commonalities and differences between cultures and will be helpful to identify developmental sequences that differ from norms based on WEIRD societies.

### Goal 3: Outsourcing of data collection

Collecting longitudinal data from different domains and from children aged 0 to 6 requires an enormous effort and is resource-intensive. With the APP, data collection is outsourced to the caregivers of the child. This approach does not come without challenges, which will be addressed in greater detail in the “4” in the 4 below. Caregivers experience their children’s behavior in more instances and more varied situations than a laboratory setting can establish. These different contexts might support the observation of the emergence a new skill. Several features were implemented to facilitate caregiver evaluation: Caregivers receive packages of questions that fit the child’s current age range in which the developmental steps usually occur. The questions are enriched with information about possible variations of the observable behavior to facilitate answering the questions. Besides this standard procedure, it is of course possible to answer questions that are not in these packages. Like this, the predefined selection of questions, which is based on the mean age of development, does not restrict answering questions that are outside of this age window. Further, the questions are complemented with additional information about the particular behavior and how it is integrated into children’s development from a broader perspective. This helps caregivers to evaluate whether or not their child already shows a certain skill or not.

To sum up, with the APP, caregivers are provided with a scientifically substantiated tool to document the development of their children between birth and the age of 6 years. It is designed to be intuitive and easy to use to facilitate continued and sustained documentation of development. The developmental steps and milestones are scientifically corroborated and have been tested for reliability by comparing them to standard instruments (see “4”, below).

In the following sections, we describe the different APP scales in more detail. We will explain the major participant target group of the APP, the construction of the different scales, ethics and data security, technical specifications, and provide details on the psychometric properties of the APP.

## Scale protocol

### Participants: Target group

The target group of the APP are caregivers of children between 0 and 6 years. We conducted a survey among 799 Swiss caregivers who already participated with their children in one or more studies of our research unit. The results showed that > 85% of the caregivers would like to use a digital developmental diary app and > 95% of these would agree to share the data with the research unit. In general, caregivers seem to be open-minded to modern media and a substantial number of caregivers is willing to use the APP and share the collected data increasing the potential for acquiring data from a sample large enough to make reliable conclusions.

### Content: Domains and items

The questions implemented in the APP target the main domains in early childhood development: cognitive, language, motor, and social-emotional skills. To obtain a comprehensive picture about the context of each individual child’s development, questions about caregiver education, country of birth, family constellation, language exposure at home and in childcare, etc., are included. An overview of all questions is available in the Open Science Framework (OSF; https://osf.io/ar7xp/).

### Construction of items

To be included in the APP, items had to fulfill two main characteristics: On the one hand, developmental skills assessed within the APP need to be scientifically relevant. That is, the skills have been documented in scientific papers on infant and child development or are included in diagnostic tools to assess the development of the skills of a child at a given age. On the other hand, the APP has to account for the fact that the questions are not answered by trained experts but by caregivers who might not be familiar with the jargon of developmental psychology. Accordingly, the assessment of skills needs to be tailored in a way that it can easily yet still reliably be performed by the caregivers, independent of their language skills and educational background. That is, for all scales, the items were formulated so that they (a) are easy to understand and imply face validity, (b) refer to the child’s observable behavior and do not require implicit measurements, (c) can be clearly, objectively, and reliably answered by the caregivers’ observation alone (avoiding sophisticated measurement techniques), (d) refer to materials which can be found in a usual household, and (e) still ensure scientific precision.

The construction procedure included the following three steps: 1) We started with a comprehensive literature search collecting skills that typically develop in the first 6 years of life in the domains of cognitive, language, motor, and social-emotional development. 2) All skills identified were evaluated with regard to whether it was possible to formulate a question and corresponding answer options that are scientifically relevant, precise, and unambiguous and at the same time feasible and understandable for laypeople. This initial collection of potential items comprised 34 items on cognitive development, 194 items on motor development, and 245 items on language development. 3) From these preliminary items, we created a first set of ‘pre’-questionnaires and asked caregivers of children between 3 and 78 months (*n* = 1397; *n*_*girls*_ = 657, *n*_*boys*_ = 739, *n*_*other*_ = 1, *M*_*age*_ = 464 days, *SE* = 526 days) to fill them out. Caregivers additionally provided feedback on whether a particular item was easy or difficult to assess or ambiguous in its formulation. Based on this feedback, 17 items were excluded from the final APP scale. Including the items of the social-emotional scale that were adapted from existing scales (e.g., the Infant Behavior Questionnaire - Revised (IBQ-R), Gartstein and Rothbart, 2003, see section “Social-Emotional Scale”, below), this process resulted in a total number of 630 items, see Table 1. The particular construction of the items in the four domains (*Scales*) is described in more detail in the following sections.

**Table 1:** Number of items per domain that were included in the final version of the APP

Domain	Number of items
Cognition	34
Language skills (Syntax, Grammar)	157
Motor	176
Social / Emotional	151
Demographics	24
Total	630

#### Cognitive scale

The items of the cognitive scale are grouped according to the following constructs: sensori-motor development, problem-solving, and numerical and categorical knowledge. The 19 sensori-motor items include questions on children’s object exploration and manipulation, reaching, attention, pointing, imitation, and pretend play. The nine problem-solving items assess the children’s object permanence, means-end behavior, memory, and mastery of new problems. The six items on numerical and categorical knowledge include questions on children’s counting abilities, color-naming skills, and knowledge about object sizes and physical laws. For item construction, the cognitive scales of existing instruments such as Bayley Scales of Infant Development (Bayley, 1993), the Intelligence and Development Scales - Preschool (Grob et al., 2013, IDS-P;), and the Griffiths Scales of Childhood Development (Green et al., 2016) were screened and served as a basis for item selection. One item was created by the authors. It describes a behavior that is commonly observed by caregivers and considered as a milestone in development but was not found in any developmental scale (CG34: “Can your child tie his/her own shoelaces?”). Each item sketches a concrete behavior or instructs to provoke a certain behavior. For details on answer options and examples of items, see Appendix.

#### Language scale

The following skills were implemented in the language scale: early pre-verbal, morphological, and syntactical skills as well as pragmatic skills. The 16 early pre-verbal skills include cooing, babbling, and the production of gestures such as pointing. The morphology scale consists of 23 items. It includes the flexion of adjectives, nouns for plural, and verbs for past and present tense. It further includes fusion of articles and pronouns or prepositions. The syntax scale comprises 65 items on the combination of clauses using conjunctions and relative clauses, Wh-questions, indirect speech and conditionals. To assess pragmatics skills, we implemented the Orion’s Pragmatic Language Skills Questionnaire (e.g., Ghahari et al., 2017), which assesses nonverbal communication, language production, conversational skills like topic maintenance and turn taking, speech conventions, and peer skills in 53 items. For all morphological and syntactical skills, we created prototypical sentences in which the target morphological flexion or syntactic construction word were highlighted. The sentences include every-day topics like caregivers working, children visiting playgrounds, reading books, etc. The words used in the prototypical sentences to express these topics are all early acquired (in the first 2–3 years) by children as cross-validated with the MacArthur Bates Communicative Developmental Inventories (Fenson et al., 2007). For details on answer options and examples of items, see Appendix.

#### Motor scale

The motor scale includes fine- and gross-motor skills. The 78 fine-motor items include visual-motor integration, grasping, and graphomotorics. The 98 gross-motor items include stationary motor skills, locomotion, and object manipulation. Item construction was geared towards existing scales such as the Peabody Developmental Motor Scales: Second Edition (Folio & Fewell, 2000, PDMS-2;) or the Bailey Scales of Infant Development: Second Edition (Bayley, 1993, BSID-ii;). Scales were screened and served as a basis for the decision regarding which items to include in the diary. For all identified motor skills, we created items that describe important motor milestones. For details on answer options and examples of items, see Appendix.

#### Social-Emotional Scale

The social-emotional scale includes measures of infants’ and children’s temperament and attention as well as their Theory of Mind (ToM). Child temperament is considered stable over time and a personality trait (Goldsmith & Campos, 1982; Rothbart, 1981; Zwickel, 2009; Thomas & Chess, 1977). Therefore, it is assumed that temperamental characteristics remain relatively stable within and across the first years of life (Bornstein et al., 2019; Carnicero et al., 2000; Pedlow et al., 1993; Peters-Martin & Wachs, 1984; Rothbart et al., 2000; Rubin et al., 2002). Therefore, unlike the items of the other scales, the items in the social-emotional scale are only asked at one point in time per scale and do not follow the AoA approach. Because ToM is often considered as not being stable, in the next version of the APP, repeated presentations of particular questionnaires will be implemented.

To assess the children’s social-emotional development, we included four scales measuring attention, early temperament, and social-cognitive development between the ages of 3 months and 6 years: 1) The Infant Behavior Questionnaire for infants aged 3 to 12 months (Gartstein & Rothbart, 2003, IBQ-R,). 2) The Early Childhood Behavior Questionnaire (Putnam et al., 2006, ECBQ,) for children between 18 and 36 months (Putnam et al., 2006). 3) The Children’s Behavior Questionnaire (CBQ) for children 3 years and older (Rothbart et al., 2001) that is suitable for the age range between 3 and 7 years. 4) The Children’s Social Understanding Scale (Tahiroglu et al., 2014, CSUS,) to assess children’s ToM. For details on the measures and answer options, see Appendix. For detailed information about the validity, and the reliability, we refer to the original publications mentioned.

## Specifications of the APP

In the following, we first provide information about data security, storage, and ethical approval, followed by technical details about the programming structure and set of the APP.

### Ethics and data security

#### Ethics approval and informed consent

The study protocol and the procedures were approved by the local ethics committee (Reference Number 20.6.5) and are in accordance with the ethical standards of the 1964 Helsinki Declaration and its later amendments. Caregivers are, for example, free to stop using the APP at any time without giving reasons for justification. All caregivers who intend to use the APP provide informed consent. No incentive other than the free use of the APP is provided to the children and their caregivers by the research unit Developmental Psychology at the Department of Psychology and the Jacobs Center for Productive Youth Development of the University of Zurich (henceforth referred to as *HOST*). When registering for the APP, a user explicitly agrees to the data processing as set out in the Terms of Use for the APP and the Privacy Policy by the University of Zurich (UZH). The HOST will continuously refine the APP. At some instances, this will lead to changes in the data processing by the HOST. Users will be notified of such changes in an appropriate manner (e.g., at the next login).

#### Data security and data protection

After installation, declarations of consent under data protection law are obtained and a declaration is made as to which data are shared with the research unit “Developmental Psychology” at the UZH and which is stored locally on the device but not forwarded to the server. All non-local data are sent via authentication tokens to a virtual server hosted and maintained by the IT Services of the UZH. Only UZH staff responsible for the maintenance of the server, the programmers for update functions, and authorized staff of the Department of Psychology and the Jacobs Center for Productive Youth Development at the UZH have access to the data. The data security strategy has been approved by the Data Security Office of the UZH and the Data Security Office of the Canton of Zurich / Switzerland (https://www.zh.ch/de/politik-staat/datenschutz.html). The data protection declaration can be viewed under https://t.uzh.ch/1dA. Cooperating research units can be granted access to parts of the data if they sign a data delivery contract with the HOST and when they have received a declaration of consent from the participating caregivers. All information about data protection is available on https://osf.io/jxspz/.

#### Data shared with the researchers

Data transmitted to the HOST is restricted to the information related to the questions asked (see https://osf.io/ar7xp/). Other data are collected solely within the APP and not synchronized with the HOST. This includes the e-mail address of the user, the name of the child, any photo or video material collected, any individual comments on specific developmental steps, own entries for personal events. These data are stored locally and encrypted on the caregivers’ own mobile device. The HOST has no access to these data.

### Technical specifications

#### Operating systems

Front end and back end of the APP have been programmed and are maintained by the companies Hybrid Heroes GmbH (Berlin, Germany, http://www.hybridheroes.de) and Smartcode (Zürich, Switzerland, http://www.smartcode.ch) as a hybrid app that works for the operation systems iOS and Android.

#### Graphical User Interface (GUI)

**Fig. 1:**
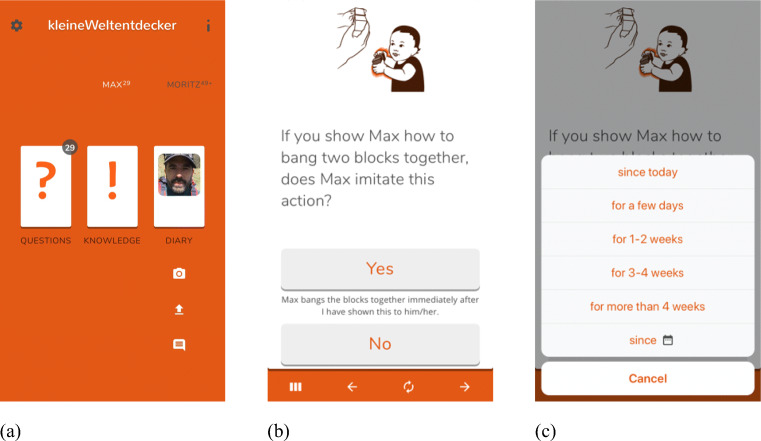
Depiction of the APP navigation: (a) Home screen of user navigation, (b) item and answer options, (c) options to indicate the time since when a child shows a particular skill

The home screen of the APP includes the following sections (see Fig. 1): 1) *Settings*: Here, basic settings can be adjusted such as the frequency and time of push notifications to inform caregivers that new questions are available for the APP, username and password, and whether or not the development of one’s own child shall be compared with the available norm values. 2) *Questions*: Caregivers are provided with the specific questions/items about developing skills in the four domains. All items and milestones are illustrated with pictures. The visual appearance of the items is based on a stack of cards. Each card contains a question about a particular skill on the front side. Swiping the card to the left reveals the next card and item. A swiping movement to the right brings back the previous item. Each card can be flipped over to reveal the section 3) *Knowledge* on the back side that includes information about the skill at question and its typical development. 4) *Diary*: For the cognition and the motor scales, caregivers see the acquired skills of their children with the corresponding date of attainment 1. Caregivers who indicated in the Settings that they wished to compare their child’s data with the available norm data can access this norm distribution derived from the whole population of children included in the APP. In the Diary section, caregivers can also add individual personal events that are not included in the set of questions (e.g., the appearance of the first tooth, the first day at the nursery, birthdays, etc). In this section, caregivers can furthermore upload pictures to enrich their diary. These individual personal events and pictures will not be shared with the HOST (see 4). 5) *Further options*: Further pages contain core data of the children (date of birth, sex).

#### Scientific illustrations

**Fig. 2:**
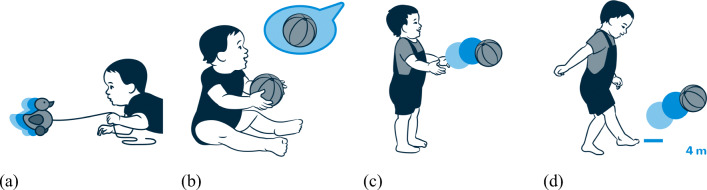
Depiction of the four age groups: (a) An infant at 4 months, (b) an infant at 12 months, (c) a toddler at 24 months, and (d) a preschooler at 48 months

**Fig. 3:**
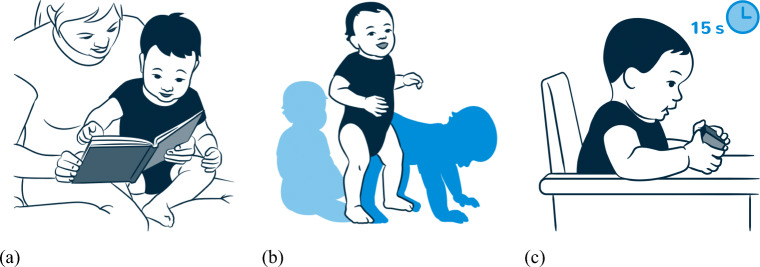
Exemplary Illustrations: (a) Child in planar style, adult in linear style, (b) movement of a child while moving from sitting to free standing, (c) additional information depicted by the duration in s

A scientific illustrator (Nadja Stadelmann, http://www.nadjastadelmann.ch) created illustrations for all items to visualize the corresponding skills. These illustrations visualize the domain (demographic information, socio-emotional skills) or a concrete developmental skill. She developed illustrations for four children of both sexes in different ages: at 4, 12, 24, and 48 months. Exemplary illustrations are shown in Fig. 2. The children are depicted using a planar style and the caregivers are depicted using a linear style. This resulted in a strong focus on the child’s behavior. To illustrate the movements, single movement steps are color-highlighted using hue saturation lightness (see Fig. 3 (b)). In some illustrations, the order of steps was accompanied with coloured arrows or numbers (see Fig. 3 (c)). In the APP, the user can assign one of eight colors to a child. The illustrations are using this basic color in combination with the hue saturation color gradation.

#### Answer options

First, caregivers answer on a dichotomous scale whether their child has attained the skill (Yes) or not (No). If caregivers indicate “Yes”, they are further asked to indicate since when the child mastered the skill. The following options are available: “since today”, “for a few days”, “for 1–2 weeks”, “for 3–4 weeks”, “for more than 4 weeks”, “since...(choose exact date)”, see Fig. 1. For some of the scales (e.g., the language scale, the social-emotional scale), the answer format deviated from this general procedure, see Appendix for more information. For each item, the questions and the answering options are presented in combination with information about the qualitative criteria of the item and the represented skill. The wording depends on the respective items. For the small “experiments” the items ask whether “my child does x”?. For other skills, the items ask whether the child is in principle able to do x (e.g., “can stand on one leg”) because the child does not always stand on one leg but might have shown this behavior already.

### Procedure

#### Caregiver information to milestones

For all items in the final APP scale, we created informative content about the skills. We summarized precursors and the development around the milestone and provided examples and contextual information. Furthermore, advice and inputs are given on how to foster developmental progress and which training would best fit this developmental phase.

### Languages

The APP is currently available in four different languages (German, French, Italian, British English). This includes three of the four languages spoken in Switzerland (except Rhaeto-Romance). Caregivers can choose in which language to use the APP. The range of languages can be expanded at any time; researchers around the globe are welcome to contact the authors.

#### Prompts and repetition of items

After installation, caregivers are prompted via push notifications periodically in time intervals between “once a week” and “once a month” to answer a short set of items about their children’s development. The time interval can be selected in the Settings section (see 4). It is possible to answer items at any time. If the caregivers respond with “No” to a certain item, this question will be repeated after a period of two weeks. The APP currently selects the items based on the earliest possible time (age in days) at which this skill was shown in a child from the data coming from the norm sample within the app. With this approach, children who have a comparably early AoA are not missed. To not miss children who have later AoAs, the questions are repeated until the caregiver indicate that the skill has been observed.

## Psychometric properties of scale

In the following, information about the psychometric properties of the APP scales is provided including objectivity, reliability, construct validity, and criterion validity. We report the psychometric properties for the cognition, language, and motor scale (except for the pragmatic language skills assessed by the Orion’s Pragmatic Language Skills Questionnaire, Ghahari et al., (2017)). The psychometric properties of the social-emotional scales are well-documented in the respective publications mentioned above.

### Participants

In the sample used to assess the psychometric properties, we included all APP data points provided by the caregivers until the date of data extraction (11 March 2020). The data were filtered for outliers and test users using the following exclusion criteria: 1) children were older than 6 years, 2) caregivers were younger than 20 years or older than 55 years^2^, 3) caregivers provided a highly unlikely birth country (e.g., Antarctica), 4) caregivers answered fewer than ten questions, 5) the AoA of a skill was before the birth of the respective child^3^. The original sample consisted of 5067 children. The application of the filtering criteria resulted in a final validation sample of 2385 children (1112 girls, 1265 boys, and eight children for whom caregivers chose ‘other’ as indication of sex). The mean age of the children at the date of data extraction was *M*_*children*_ = 791 days, *SE*_*children*_ = 11 days. In this validation sample, the APP was used by 1984 mothers, 294 fathers and 16 other caregivers, 91 did not answer this question. The mean age of the APP user at the date of extraction was *M*_*user*_ = 36 years, *SE*_*user*_ = 0.08 years.

For construct validation, we invited caregivers (*N* = 256) who filled out the ‘pre’-questionnaires (see 4) for their children to participate in a lab study with their children. We compared caregivers’ answers in the ‘pre’-questionnaire to their child’s performance in lab-based standardized tests. The validation sample for the cognitive scale included 74 children (*n*_*girls*_ = 36, *n*_*boys*_ = 38, *M*_*age*_ = 734 days, *SE* = 42 days). validation sample for the motor scale included 97 children (*n*_*girls*_ = 46, *n*_*boys*_ = 51, *M*_*age*_ = 873 days, *SE* = 63 days). The validation sample for the language scale included 85 children (*n*_*girls*_ = 38, *n*_*boys*_ = 47, *M*_*age*_ = 1480 days, *SE* = 60 days).

### Analyses plan

In the following sections, we describe the different psychometric properties of the APP scales. To analyze objectivity and criterion validity, we used different multi-level logistic regressions predicting either the AoA for the motor and cognitive items or the language scale index for language skills by domain (motor or cognition), caregiver education (mother and father), caregiver age (mother and father), app user (mother or father), sex of the child, and pregnancy week in which the child was born (see also Eq. 1 in the Appendix). Details about the specific analyses are reported in the respective sections below. To measure construct validity, we predicted children’s performance in lab-based tests with the answers of caregivers for the according items in the APP assessed via the ‘pre’-questionnaires using multi-level regressions for the motor, cognition and language scale. As a reliability measure, we assessed the internal consistency by calculating Cronbach’s *α* separately for the different scales and age ranges.

### Objectivity

To assess objectivity, we analyzed the influence of the APP users in our regression on the AoA in the motor, cognitive, and language scales. That is, we tested whether it made a difference whether the data were entered by mothers, fathers, or other users. The results showed that the factor APP user had no influence on the indicated AoA, see Tables 2 and 3.

**Table 2:** Psychometric values for the assessment of the objectivity and criterion validity for the motor and cognition items: Type III analysis of variance table with Satterthwaite’s method

	Sum Sq	Mean Sq	NumDF	DenDF	F value	Pr(>F)	Sig. Level
Pregnancy_Week_	20943	20943	1	1700.2	7.118	.008	**
Sex	517	259	2	1697.2	0.088	.916	
Age_Mother_	206250	206250	1	1699.9	70.100	< .001	***
Age_Father_	20175	20175	1	1697.5	6.857	.009	**
Education_Mother_	15525	3105	5	1696.8	1.055	.384	
Education_Father_	14553	2911	5	1693.0	0.989	.423	
APP_User_	4803	2401	2	1697.1	0.816	.442	
Domain	103	103	1	206.0	0.035	.852	

Because there were only few cognitive items, we merged items of the motor and the cognitive items in this model and included domain as a factor. There was no effect of domain. The model accounted for 98.28% of the variance

**Table 3:** Psychometric values for the assessment of objectivity and criterion validity for the language items: Type III analysis of variance table with Satterthwaite’s method

	Sum Sq	Mean Sq	NumDF	DenDF	F value	Pr(>F)	Sig. Level
Age_Days_	472779	472779	1	289.29	582.325	< .001	***
Pregnancy_week_	1589	1589	1	387.45	1.957	.163	
Sex	4751	4751	1	410.92	5.852	.016	*
Age_Mother_	95	95	1	321.50	0.118	.732	
Age_Father_	362	362	1	315.64	0.446	.505	
Education_Mother_	2053	513	4	321.49	0.632	.640	
Education_Father_	1350	270	5	336.51	0.333	.893	
APP_User_	2392	1196	2	310.82	1.473	.231	

The model accounted for 65.24% of the variance

### Reliability

For all scales, we assessed the internal consistency by calculating Cronbach’s *α* separately for the scales and age ranges. See Table 4 for an overview of the results in the single scales and age ranges. The results indicate a range between acceptable (*α* > .70) and excellent (*α* > .90) reliabilities for almost all age ranges for the domains of fine motor, gross motor, and language. Only the value for fine motor skills between 12 and 18 months was slightly below the acceptable value of *α* = .70. The reliability scores for the cognition items were less solid and mostly ranged below *α* = .60 with the exception of the age range between 3 and 6 months (*α* = 81).

**Table 4:** Internal consistency (Cronbach’s *α*) for cognition, language, fine motor, and gross motor items for different age ranges

Scale	Age Range (Months)	*α*
Cognition	3 - 6	0.81
Cognition	6 - 12	0.449
Cognition	12 - 18	0.424
Cognition	18 - 24	0.421
Cognition	24 - 36	0.575
Cognition	36 - 48	0.334
Language	24 - 36	0.985
Language	36 - 48	0.982
Language	48 - 72	0.982
Fine Motor	3 - 6	0.918
Fine Motor	6 - 12	0.831
Fine Motor	12 - 18	0.653
Fine Motor	18 - 30	0.742
Fine Motor	30 - 44	0.817
Fine Motor	44 - 72	0.835
Gross Motor	3 - 6	0.9
Gross Motor	6 - 12	0.889
Gross Motor	12 - 18	0.738
Gross Motor	18 - 30	0.748
Gross Motor	30 - 44	0.755
Gross Motor	44 - 72	0.812

### Validity

#### Construct validity

Each scale was validated for different age groups. We used the pre-questionnaire (see 4) to assess caregivers’ answers to items of the APP and compared them to children’s behavior in the corresponding items of existing scales (see below for details on which scales were chosen) using logistic regressions. We followed the procedures and scoring guidelines of the existing scales.

For the motor scale, we tested children’s motor skills with the motor items of the Bayley Scales for Infant and Toddler Development III (Bayley, 2005, BSID-III,) up to 42 months and with the Peabody Developmental Motor Scales (Rhonda Folio and Fewell, 2000, PDMS,) for children older than 42 months. For the cognitive scale, we used the items of the cognitive scale of the BSID-III (Bayley, 2005). For the language scale, we used the “Test zum Satzverstehen von Kindern” [*Test of Sentence Understanding of Children*] (Siegmüller et al., 2011, TSVK,) to assess syntactic and morphological skills and the Peabody Picture Vocabulary Test (Dunn & Dunn, 2007, PPVT-4,) to assess children’s vocabulary size.

Multi-level logistic regressions were calculated to predict children’s motor and cognitive performance in the lab (i.e., whether or not children showed the respective behavior when assessed in the lab) for each item individually by the answers the caregivers provided in the pre-questionnaire of the APP. The regression controlled for children’s age and the time span between the dates when caregivers answered the APP question and when their child was tested in the lab. For the *Motor scale*, the caregivers’ answers significantly predicted the children’s lab performance, *estimate* = 1.671,*SE* = 0.196,*z* = 8.548,*p* < .001, as well as age, *estimate* = 0.093,*SE* = 0.027,*z* = 3.476,*p* < .001. The model accounted for 51.62% of the variance. This was not the case in the *Cognitive scale*, where caregivers’ answers neither predicted children’s performance in the lab, *estimate* = 0.212,*SE* = 0.427,*z* = 0.496,*p* = .620, nor age, *estimate* = 0.019,*SE* = 0.030,*z* = 0.632,*p* = .527.

For linguistic skills, we calculated the grammar scale index, summing up the usage frequencies of each item, that is, how often each item occurred in a child’s language production. With this grammar scale index, we predicted the total PPVT score and the TSVK score collected in the lab. Both the PPVT and the TSVK score were calculated following the instructions in the corresponding manuals. We ran linear regressions on the TSVK and the PPVT scores controlling for the time span between both tests, *M* = 58 days, *SD* = 27 days. Results showed that the grammar scale index significantly predicted the TSVK score, *estimate* = 1.450,*SE* = 0.469,*z* = 3.092,*p* = .003, and the delay *estimate* = − 0.065,*SE* = 0.021,*z* = − 2.999,*p* = .004. The model accounted for 27.05% of the variance. Similarly, the grammar scale significantly predicted the PPVT score, *estimate* = 18.321,*SE* = 7.451,*z* = 2.459,*p* = .024. However, we did find no effect of delay, *estimate* = − 0.496,*SE* = 0.546,*z* = − 0.907,*p* = .376. The model accounted for 41.42% of the variance. In sum, the standardized lab test performances were predicted by caregivers’ answers to the language scale questions of the APP, which shows excellent content and predictive validity.

#### Criterion validity

We investigated criterion validity by predicting the AoA outcomes and language scores with factors that typically effect development. Here, we tested the pregnancy week a child was born, child’s sex, age and education of father and mother, and the APP user (i.e., whether father, mother, or another caregiver provided the data) and entered them into the model. The results are shown in Table 2 (Motor scale and cognitive scale), and Table 3 (Language scale). For the motor and the cognition scale, we found that AoA was predicted by the pregnancy week with births in earlier pregnancy weeks being associated with later AoA, and effects of caregiver age, with older caregivers showing later AoA, an effect that was stronger for mothers’ age than fathers’ age. We found no effects for domain (cognition, motor), child’s sex, caregiver education, and APP user. For the language scale, we found that boys were evaluated as having poorer language skills than girls and language skills increased with age, see Table 3. In sum, factors that typically effect development such as pregnancy week or gender also influenced the scores that were obtained by the caregivers. We therefore conclude that the APP has sufficient construct validity.

Our analyses of the psychometric properties of the APP indicate a sufficient objectivity, reliability, and validity for the motor and language scales. For the cognition scale, reliability and validity measures need to be improved in future versions of the APP by editing, including, or excluding individual items (see 4).

## Initial and preliminary findings

To further substantiate the validity of the items used and the general method of ambulatory assessment based on a digital developmental diary, we present some initial findings of what can be measured and whether and how previously reported findings are replicated. First, we present some data that describe the sample drawn for the current purpose (see 4, date of data extraction: 11 March 2020). Second, we present a preliminary replication of the relationship of non-verbal and verbal communication skills (see 4, below).

### Descriptive data and demographics

Caregiver answered on average 75 questions, ranging between 10 and 285. The mean duration between registration and last usage (i.e., the mean length of usage) is 4.32 months, ranging between 1 months and 16 months. On average, caregivers filled in questions on 2.44 different days per month, ranging between 0.15 and 16 days. Caregivers answered questions on average every 21 days (*M* = 21.43 days, *SE* = 0.31). Per day, caregivers answered on average 3.01 questions (SE = 0.06) questions in cognitive development, 12.53 questions (*SE* = 0.22) in motor development, 7.06 questions (*SE* = 0.25) in language, 2.89 questions (*SE* = 0.12) in social-emotional development, 3.27 questions on physical measures and 10.7 questions (*SE* = 0.1) on background variables.

Further, as an example of geographical distribution, we collected data on the countries of birth and living, see Table 5. Currently, most of the users live in Switzerland (54*%*) and Germany (38*%*), in total 88 countries of residence were indicated. Finally, as one example for demographic variables, caregivers are asked to indicate how their child was born, either via natural birth or via Caesarean section. The Swiss Federal Statistical Office (Statistik, 2020) reports for the year 2017 that almost one-third (32.3*%*) of all newborns (*n* = 85.990) in Switzerland were be born by Cesarean section. The data collected in our APP reveal the percentage of 32.4*%* (*n* = 790 out of *N* = 2437). The two percentages are almost identical, with no statistical difference between them, *χ*^2^ = 0.009,*p* = .931. While we don’t claim that the data generated by the unsupervised APP use are representative for the population, it seems that they approximate population statistics in such key variables.

**Table 5:** Number of users (total, mothers, and fathers) of the 15 countries with the largest numbers of users

Country	Total	*n*(*Mothers*)	*n*(*Fathers*)
Switzerland	2472	1227	1245
Germany	1827	918	909
Austria	68	35	33
Italy	52	31	21
Poland	35	12	23
United States	23	12	11
Turkey	22	14	8
Russia	21	9	12
United Kingdom	18	15	3
Romania	18	8	12
Spain	18	12	6
Bosnia	17	6	11
France	15	9	6
Kazakhstan	15	2	13
Kosovo	13	7	6

### Communicative skills

**Fig. 4:**
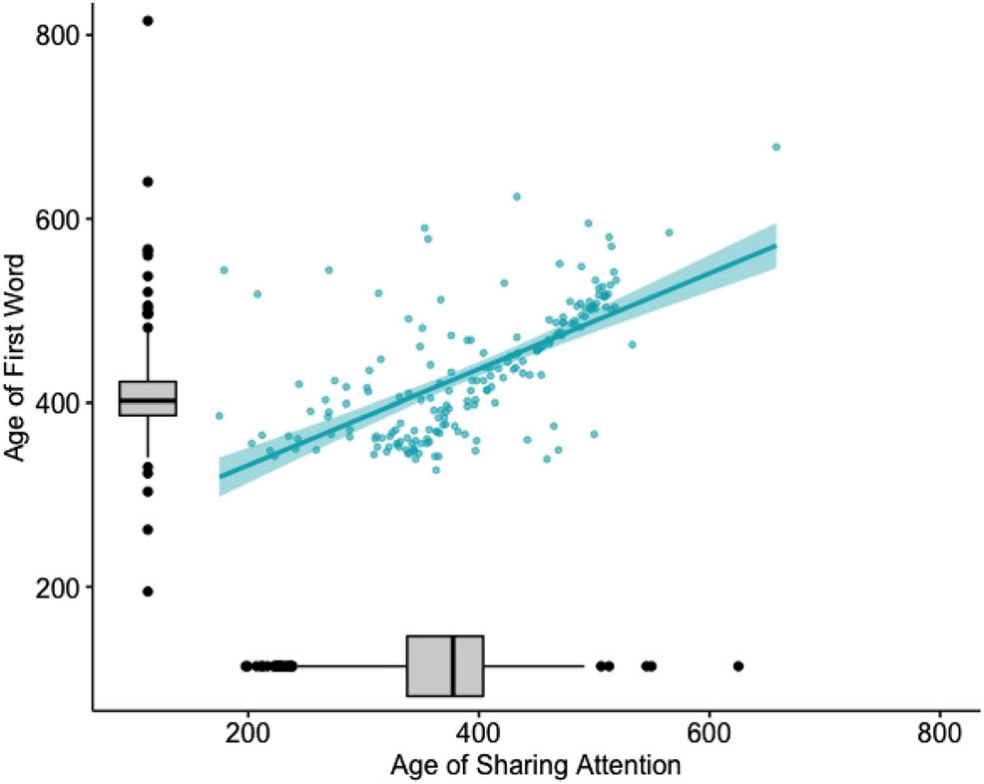
Relationship between AoA of Sharing Attention as an indicator of early joint attention ability and the AoA of First Word as an indicator of early language skills. Individual data points (*blue*) and box plots are illustrated

Finally, we present data on a developmental psychological aspect of the APP data: The development of the interrelation between non-verbal and verbal communication. Previous research in laboratory settings reported a longitudinal relation between joint attention and language development (Farrant & Zubrick, 2012; Morales et al., 2005). Children who had low levels of joint attention during infancy were significantly more likely to have poor receptive vocabulary around age of 5 (Farrant & Zubrick, 2012). Also, children with lower scores in pointing at the age of 12 months (pointing only with open hand but not yet with index finger) were at risk for language delay 1 year later (Lüke et al., 2017). The analysis of a subset of infants (*n* = 198, *n*_*girls*_ = 97, *n*_*boys*_ = 101) taken from the APP data indicated that the onset of early joint attention ability (i.e., child is looking from an object to caregivers and back) significantly predicted the age at which infants spoke their first words, β = 0.51,*p* < .001,*F*(3,194) = 50.28,*p* < .001,*R*^2^ = 0.44. There was no main effect of infants’ sex, β = − 21.81,*p* = .563, nor an interaction of sex and early joint attention, β = 0.06,*p* = .495. Infants who showed joint attention earlier in life also spoke sooner (see Fig. 4). This is in line with the above-mentioned previous findings that infants’ developing non-verbal social-cognitive skills are longitudinally related to their emerging language skills.

## Discussion

In this paper, we present a new tool for the assessment of children’s development from birth to the age of 6 years. Via the use of a smartphone-based developmental diary application (the kleineWeltentdecker App, referred to as the APP), caregivers can track the emergence and the development of their children’s skills in four major developmental domains. The empirical validations of the reliability of the procedures with children of different age groups have (except for the cognition items) confirmed the robustness of the different measures implemented in the APP. In the following, we discuss the psychometric properties, the goals, and the challenges of the APP.

### Psychometric properties

The assessment of the psychometric properties resulted in an overall positive outcome. The high *objectivity* of the data is indicated by the fact that no differences were found between the AoAs and the usage frequency between mothers, fathers, and other caregivers, which was the case in all scales.

#### Reliability

The assessment of *reliability* resulted in mixed findings and the reliability critically depended on the scale tested. Reliability was excellent for the language scale and good for the motor scale. There is room for improvement with respect to the cognition scale, for which the reliability was generally below acceptability.

There are several potential reasons that can explain the non-optimal results for the cognition scale. First, the scale consists of fewer items (*n* = 34) compared to the other scales (all *n* s > 100), which might result in a larger variability of the results. Second, the range of tested skills is relatively broad and thus heterogeneous. It ranges from simple sensori-motor items to more complex tasks on memory or problem-solving. Third, and probably most important, the items often involve the instructions for caregivers to conduct a little “experiment” with their children. For example, basic memory functions are assessed via the following item: “Try this little experiment: Put 3 pairs of shuffled memory cards picture-side up on a table in two rows. Ask [Child name] to remember where each card is. Then, turn over the cards one by one so that the pictures are no longer visible. Now ask [Child name] where the paired pictures are, one by one.” (Item CG32). While the instructions are formulated as easy-to-understand, caregiver-friendly, and unambiguous as possible, there is still room for variation in how exactly caregivers perform these experiments and how the child’s behavior is interpreted. Also, the relatively high cost for the caregiver to perform the task (e.g., getting up and searching for memory cards) might have prompted a positive response even though the skill had not yet been developed. Other instruments that assess children’s cognitive development require trained examiners to perform the assessment. Findings from citizen science research are helpful to shed more light on this increase of variability: On the one hand, citizen scientists can perform collections of valid basic data even when given only a brief training (Darwall & Dulvy, 1996; Evans et al., 2005; Fore et al., 2001; Graham et al., 1996). On the other hand, data validity is decreased when citizen scientists are confronted with more complex questions and observation tasks, such as observations in astronomy (Balcom, 2015). In general, without proper training in experimental protocols, citizen scientists (such as the caregivers who use the APP can be compared to) are more likely to introduce variability into their data (Eaton et al., 2002; Danielsen et al., 2005). Applied to the present set of cognitive items, it might be the case that they are in general more difficult to evaluate than the motor or language items. To conclude, the data collected with the current cognition items are not yet as reliable as the other scales. Further developments are required to improve this scale.

#### Validity

Our analyses on construct and criterion validity yielded no effects of caregiver education on the data. This indicates that caregivers of all educational backgrounds respond similarly to the questions. Caregiver education is only a rough estimate for a more global assessment of caregivers’ socio-economic status (SES). We will, therefore, evaluate whether a more differentiated assessment of SES (e.g., asking for income and other aspects) is required and likewise feasible and accepted by caregivers. The present data showed that children’s sex had a significant effect on their language development. Girls generally had earlier language AoAs than boys. This finding is well established in the field: Girls produce sounds and use words at an earlier age, have larger vocabularies, greater grammatical complexity, and read sooner than boys (Bornstein et al., 2004; Stolt et al., 2008; Reilly et al., 2019; Miller & Halpern, 2014; Lisi et al., 2002; Lange et al., 2016). Interestingly, the AoA was related to the caregiver age. Children of older caregivers had later AoAs. Previous research reports that caregiver income related to better problem solving and language scores (Yeung & Linver, 2002) and that caregiver job loss had an impact on their children’s performance in school (e.g., Rege et al., 2011; Stevens & Schaller, 2011), an effect that seems already visible even before children enter school (Mari & Keizer, 2020). Caregiver income and their SES increase with caregiver age (e.g., Featherman et al., 1988; Mclanahan, 2004; Powell et al., 2006; Ross & Mirowsky, 1999). Children from older caregivers should therefore have earlier AoAs than children from younger caregivers. In light of this tendency, the present data are in contrast with this previous data. However, the analysis of the demographics indicated that the general level of education of the caregivers using the APP was relatively high and variability was relatively low. Previous research shows that caregiver education and children’s outcome are considered at a bivariate level only, the relationship can be curvilinear and disadvantageous for children with comparatively young or old caregivers (e.g., Powell et al., 2006). However, when considering additional factors such as SES or family structure, the pattern typically becomes linear and caregiver age becomes positively linked to child outcomes. These aspects require further attention as the amount and the quality of the data increase. In general, the results of the analysis of the psychometric properties are promising. This is particularly the case for the motor and language scale whereas the results for the cognition scale are more heterogeneous.

### Goals and challenges

In the Introduction, we formulated three major goals. We aim 1) to establish a comprehensive data set of child development, 2) to have tool that accounts for the variability on development across cultures beyond WEIRD countries, and 3) to outsource data collection to caregivers. In the following, we discuss how the APP can help researchers to reach these goals and the challenges that have yet to be met.

#### Goal 1: Establish a comprehensive data set of child development from age 0 to 6, within and across individuals

With the APP, we aim to obtain data that inform about the variability of behavior and its development in relation to contextual aspects. The APP measures children’s competencies in the cognitive, language, motor, and social-emotional domains of development. Furthermore, questions on children’s culture, SES, and language background offer information on their environment. The analysis of data acquired by the APP is not limited by time-consuming processes of manual coding of behavioral data. For the analysis, it does not make a big difference whether the data set includes 30 or 30,000 participants. The data of the APP will provide information that goes far beyond what has been called the “taking snapshots of developmental outcomes” approach (Adolph et al., 2008; Caspi et al., 1996), and has the potential to substantially increase our understanding of developmental processes. The APP uses an Age-of-Attainment (AoA) approach (Eaton et al., 2014) that is centered on the date of emergence of a developmental skill. Individuals differ in their AoAs. This particular variability is of key interest because it provides evidence about how long it takes individuals to reach a particular skill and to move to the next skill. That is, it allows evaluating individual differences in the chronological AoA and the temporal distances between the AoA of two (or more) different skills. Eventually, this allows a detailed description of individual developmental trajectories and the identification of the interrelations between skills within and across domains. These descriptions of developmental trajectories are essential for the advancement of theories about children’s development and the acquired data will help to significantly increase our understanding of developmental change in childhood.

#### Goal 2: Account for the variability on development across cultures

Previous research in developmental psychology has to a large extent been based on WEIRD populations, which has recently been criticized (e.g., Nielsen et al., 2017). Therefore, variability of behavior and development is underestimated. The approach of the APP allows moving beyond sampling from highly homogeneous (often WEIRD) populations to a sample of large variability with respect to cultures and social contexts. Data collected with the APP allows comparing development within and between cultures and drawing conclusions from highly diverse samples. This approach helps to fulfil the plea raised by, for example, Nielsen and colleagues (2017) that a “complete understanding of the ontogeny and phylogeny of the developing human mind depends on sampling diversity” (p.32), which receives further and increasing support by numerous other researchers (Clegg & Legare, 2016; Henrich et al., 2010c; Legare & Harris, 2016; Nielsen & Haun, 2016; van Schaik & Burkart, 2011). This sort of data as measured by the APP is essential to broaden our theoretical understanding about which aspects of the development of skills and traits are universal and which culture-specific.

#### Goal 3: Outsourcing of data collection

With the APP, we outsource data collection of longitudinal high-density data to caregivers. With this, we aim to reduce the enormous personal and financial resources associated with such data collection. Moving data collection from the controlled setting of a laboratory to the “real, noisy world” and from the hands of trained and experienced experimenters to the caregivers comes with several challenges. Previous research suggests that, at least for motor and language skills, caregiver checklist diaries are concordant with experimenter home visits (e.g., Bodnarchuk & Eaton, 2004). The present validation of the motor scales converges with this finding, but given the not optimal results from the validation of the cognitive scales, some skepticism regarding the reliability of caregiver reports might remain. In the following, we present three current challenges and the approaches of how they have been or will be addressed in future refinements of the APP. The solutions might not be final, and experience and time will provide a more detailed view on how and how not to address the challenges satisfactorily.

#### Challenge 1: Infrequent use of the APP

Research designs that use online surveys and smartphone applications are attractive. They come at relative low cost and offer great flexibility (LaRose & Tsai, 2014; Barrios et al., 2011; Evans & Mathur, 2005; Fan & Yan, 2010; Fricker & Schonlau, 2002; Kaplowitz et al., 2004). At the same time, they are subject to lower completion rates than conventional survey methods (Börkan, 2010; Jones & Pitt, 1999; Manfreda et al., 2008; Sax et al., 2003; Shih & Fan, 2008). It is therefore likely that a substantial number of caregivers will not use the APP regularly. This will result in a large amount of missing data. There may be ways that help to increase caregivers’ commitment. Incentives such as monetary compensation (Frick et al., 1999), loyalty points (Göritz, 2008), or sweepstakes offering of a certain monetary value (LaRose & Tsai, 2014) have been shown to increase commitment in online studies (as indicated by an increase in response rate to invitations and completion rate, but see (Göritz, 2006) for null results). For an extensive overview of psychological and data collection via the Internet, the reader is referred to the extant literature (e.g., Birnbaum, 2004; Manfreda et al., 2008; Reips, 2002; Shih & Fan, 2008). However, given the already large number of participating caregivers (> 4.000; March 2020), it is not feasible to offer any form of monetary incentive to all users.

One potential option is to offer caregivers to participate in a lottery that takes place periodically where caregivers can win a voucher that can be used worldwide (e.g., in online music or book stores). Lotteries seem to have a positive impact on participant commitment (LaRose & Tsai, 2014). Participation in the lottery could be automatized or applied as an incentive if caregivers contributed a predetermined number of data points within a given period. A second option is to implement the APP as a supplementary measure in a more controlled setting of an existing ongoing longitudinal study. In such a setting, a smaller number of caregivers who agreed to take part in a study can be motivated via incentives more easily and reminded repeatedly to answer the questions. With this approach, the usage and data of “unsupervised” caregivers can be compared to a highly controlled sample of caregivers, which will provide further insights to the interrelation between use and data quality. Two examples for this second option are first, the study Children and Digital Media (Kinder und digitale medien, 2021, KiDiM;) by the Marie Meierhofer Institute in Zurich, Switzerland. Here, APP data complements the collection of longitudinal data on children’s media use. Second, in a planned study on the relation between nutrition and cognitive development, currently prepared by the USZ Neonatology section (Natalucci et al., 2021, LEARN;), APP data will be used as a continuous measure of the children’s development between the specified measurement times. One major aim for the future will be to use the data coming from “supervised” and “unsupervised” users to identify different user behavior, its impact on the data quality, and ways to impute missing data. High-resolution data from “supervised” users will thereby serve as a basis to impute missing data of the low-resolution data of the “unsupervised” users.

#### Challenge 2: General reliability of questionnaire data

One might in general be skeptical about the reliability of the data caregivers report. They might be inclined to answer the questions about the development of their children too optimistically for reasons of social desirability. Previous research on the reliability between laboratory assessments and caregiver reports revealed inconsistent effects. Whereas caregiver scores often correlate with professional assessment, they likewise tend to be a poor predictor of infants at risk of developmental delay (e.g., Emond et al., 2005). Caregiver report and experimenter home visit observations seem to converge when assessing the development of motor skills. For example, the Parent Milestone Report Form has been shown to be a reliable and valid instrument to assess infants’ development of a number of gross motor skills via caregiver report (Adolph et al., 2008; Bodnarchuk & Eaton, 2004). Similarly, Miller and colleagues (2017) showed no differences in the evaluation of receptive, expressive language, and fine motor skills between caregiver report and direct assessment via experts. Further, laboratory tests and experimenter home visits often underestimate children’s early linguistic abilities (Bates, 1993) and adults are generally good in estimating skills. Self-reports, for example in language proficiency evaluation, converge with objective measures in adults (e.g., Marian et al., 2007). Furthermore, the current approach receives support by the fact that caregivers are with their children in many more and highly variable situations than a laboratory setting is able to establish. This makes it more likely that they observe the skill that the APP asks for and that caregivers’ assessment of their children is close in time to the first occurrence of a skill. In contrast, when caregivers are asked to assess the emergence of their children’s first words (around the age of 12 months) retrospectively, these estimates show only weak reliability that decreases with increasing age of their children (Majnemer & Rosenblatt, 1994, *r* = 0.27 at age 3 and *r* = − 0.11 at age 5,). The analysis of the APP user objectivity was generally successful, the results showed that the factor “APP user” had no influence on the AoA identified. However, one issue needs to be discussed more critically. While the reliability was evaluated by two independent observers, a caregiver via the questionnaire and a researcher in the controlled laboratory environment, this was not the case in the objectivity rating, which may have caused selection effects (e.g., fathers who are more involved in raising their children might be more inclined using the APP and might, therefore, be better observers). This is a limitation for larger conclusions concerning objectivity. However, for those caregivers who have been using the APP so far, this does not seem to be a major problem.

In general, while caregivers may be susceptible to social desirability effects, they are still likely the most reliable source to determine whether or not a skill is in their child’s repertoire (Sachse & Suchodoletz, 2008). Caregivers are in the unique position to observe and interact with their children across many different situations. This makes them likely to report the particular moment when a skill was observed for the first time. The everyday interaction of caregivers with their children is furthermore not subject to issues with child motivation and cooperation and has been established as a valuable way to quickly and cost-effectively add information important for the detection of developmental delays (e.g., Nordahl-Hansen et al., 2014). These aspects result in an increased application of caregiver reports for routine developmental screening that are particularly helpful for the identification of children at risk for developmental delays, a procedure that is in accordance with the recommendations by the American Academy of Pediatrics (e.g., Emerson et al., 2016; Johnson & Myers, 2007).

With respect to reliability, the current approach represents a trade-off between feasibility and reliability. Optimally, caregivers would answer the question about the emergence of a particular skill at several instances, repeated over several days to account for the variability of the emergence of a particular skill (Adolph et al., 2008). However, workload for the caregivers is already substantial in the current version of the APP with up to 20 questions per notification. A further increase of this number will result in a significant decrease of the number of participating caregivers. The current approach relies on the fact that caregivers are required to answer the questions about the development of their children in intervals of 1 week or 1 month. Considering these circumstances, we are confident the APP a valuable and important tool that avoids false negatives. Caregivers observe their children in a variety of different situations and, triggered by the particular question posed by the APP, might bring their children in situations in which the new skill is likely to be observed. It is important to emphasize that the present scales will not replace any diagnosis of clinical symptoms or developmental delays, which need an in-depth diagnosis of an expert psychologist and/or pediatrician.

Finally, to reliably assess the emergence of a developing skill, measuring daily fluctuations would be optimal. The APP is not designed to fulfil this purpose but to assess whether and when a child shows a skill with a defined quality. This approach is inspired by developmental assessment tools like the Bailey Scales (Bayley, 2005) and by the AoA approach (Eaton et al., 2014). The collected AoAs are snapshots, subject to fluctuations over time. However, the questions are answered by the caregivers not on the basis of a single observation at one point in time but on their everyday observation of their children. The advantage of this approach is to collect data from a large number of children in a relatively easy and convenient way and to be closer in time to the actual AoA of a skill than traditional approaches.

#### Challenge 3: Assessment of language skills

While the APP may document children’s development in the cognitive, motor, and social-emotional domain, the measures on language development are not yet comprehensively integrated. The current assessment of linguistic skills does not yet include vocabulary. In an initial version of the APP, we included this aspect with almost 2000 items being asked to caregivers. The feedback caregivers provided indicated that they became tired quickly of answering the vocabulary items due to the sheer number of questions about single words their child might or might not speak at a given point in time. For this reason, we removed the vocabulary section in the current version. One potential approach to this challenge was recently introduced by Mayor and Mani (2019). These authors presented a new methodological approach through which an estimation of a child’s vocabulary score (Fenson et al., 2007, as assessed by the MacArthur Communicative Development Inventories, CDI;) can be obtained by combining caregiver responses on a limited set of words sampled randomly from the full CDI with the information about how many children do or do not speak a particular word extracted from the WordBank database (Frank et al., 2017). The findings show that using a reduced list of only 25 words provided an accurate estimate of a child’s vocabulary size for American English, German, and Norwegian. Implementing an algorithm similar to the one used by Mayor and Mani (2019) can be one way to improve the procedure by substantially reducing the number of vocabulary items. This is a plan for future implementation.

To sum up the challenges, there are reasons that caregiver assessment will be both more and less precise than laboratory assessment. Collecting more data, asking feedback from participating caregivers, and using this information to adjust items accordingly will help to increase reliability in the future. For example, with more data analyzed, a “real” effect may become visible and the influence of individual children on the data becomes less substantial.

### Anticipated outcome and significance

Given the ubiquity of smartphones worldwide, smartphone applications increasingly serve as digital support devices. With the developmental diary application presented here, we put a portable data acquisition tool in the pocket of caregivers. The value of this approach is high (in times of the SARS-CoV-2 pandemic in which this paper was partially written, even more so). It includes longitudinal data of a potentially large-scale, population-based sample. The sample size is not restricted to areas or by limited (financial and human) resources. This approach allows to move beyond sampling from highly homogeneous (often WEIRD) populations to a sample of great variability with respect to cultures and social contexts. The data collected with the APP allow comparing development within and between cultures and drawing conclusions from highly diverse samples. Eventually, this allows a detailed description of individual developmental trajectories, and the identification of the interrelations between skills within and across domains.

With the approach presented here, data are collected from more children in more places at a higher frequency than it is possible with moderated testing, either in-person or online. The descriptions of developmental trajectories derived from these data are essential for the advancement of theories about children’s development and the acquired data will help to increase our understanding of developmental change in childhood.

## Equation used for analyses of psychometric properties

To analyze the objectivity and criterion validity of the APP, we used different multi-level logistic regressions predicting either the AoA for the motor and cognitive items or the language scale index for language skills by domain (motor or cognition), caregiver education (mother and father), caregiver age (mother and father), app user (mother or father), sex of the child, and pregnancy week in which the child was born (1).

1 $$ \begin{array}{@{}rcl@{}} &&\text{AoA/Language Index} \sim \\ &&\text{Domain}+\text{Education}_{\text{Father}} + \text{Education}_{\text{Mother}} + \\ &&\text{Age}_{\text{Father}} + \text{Age}_{\text{Mother}} + \\ &&\text{APP}_{\text{User}} + \text{Sex}_{\text{Child}} + \text{PregnancyWeek}_{\text{Child}} + \text{(1|Item)} \end{array} $$

## Appendix:

### Details on scales

In the following sections, we describe the answer options of the cognitive, language, motor, and social-emotional scale in more detail and give examples of answer options and items of each scale. All the items of all scales are available on https://osf.io/ar7xp/.

#### Cognitive Scale

##### Answer options

Caregivers are asked to indicate whether or not they observe the behavior in question. We display answer options in a dichotomous manner. The positive option is “Yes” and, to be unambiguous, relevant factors of the respective behavior are additionally outlined concisely for some items. Hence, the answer options include exact success criteria which have to be met (e.g., counting three or more objects). The negative option is “No” and, where necessary, alternative behavior which would not justify a positive choice is sketched (e.g., counting less than three objects). As in the questions for the motor scale, if caregivers indicate “Yes”, they are asked to additionally indicate since when the child mastered the skill, see Fig. 1.

##### Exemplary items

To illustrate, we exemplarily describe one item for each construct. An early *sensori-motor* item on exploration behavior asks: “Does your child play with or examine his/her fingers?”. Caregivers can answer with either “Yes, when my child is calmly lying on his/her back, my child plays with his/her hands or examines them (turning them around, opening and closing them)” or “No”. One of the first *Problem-solving* items assesses means-end behavior: “Does your child pull at flat objects (e.g., a cloth or blanket) to grasp a toy that is lying on it?”. This item has the answer alternatives: “Yes” or “No, my child moves towards the toy”. A *Numerical and categorical knowledge* item investigates children’s counting abilities: “Can your child count out 10 objects (e.g., building blocks)?”. Caregivers can answer with “Yes, my child says “one” to the first object, “two” to the second, and “three” to the third, etc.” or “No”.

#### Language Scale

##### Answer options

For each sentence of the morphological and syntactical items, caregivers are instructed to indicate how often their child produces such or a similar sentence. The questions can be answered via a five-point Likert scale (never; less than once a week; at least once a week, but not every day; once or twice a day; more than twice a day) similar to the Children’s Communication Checklist – 2 (Bishop, 2003, CCC2,).

##### Exemplary items

To illustrate, we provide some examples for items and prototypical sentences. An item for *Gestures* asks for: “Does [Child’s Name] point at objects that [Child’s Name] wants or would like to show you?”. An item for *Morphology* assesses one of the plural forms in Swiss German like this: “Holsch üsi **Jackene**?” (Engl. “Can you bring our **jackets**?”). An item for *Syntax* conjunctions includes the following sentence: “Wämmer wiiterspile **oder** wetsch es Buech aluege?” (Engl. “Do we want to keep playing **or** should we look at a book?”).

#### Motor scale

##### Answer options

For each item a dichotomous scale is used with which the caregivers are asked to indicate whether their child has attained the skill (Yes) or not (No). If caregivers indicate “Yes”, they are further asked to indicate since when the child mastered the skill, see Fig. 1.

##### Exemplary items

To illustrate, we provide some examples for typical items. *Visual-motor integration* items assess eye-hand coordination with objects: “My child can stack 5 building blocks” and without objects: “My child can clap his/her hands”. Specific *Grasping* items assess the way objects are grasped and held how a child uses his/her hands for grasping: “My child can grasp a rattle, lying on his/her back”. *Graphomotorics* items assess how the child uses pencils to draw and write: “My child imitates me drawing a line”. *Stationary motor skills* items assess body posture: “When I place my child on his/her tummy and shake a rattle, he/she rotates his head to locate the rattle”. *Locomotion* items assess movement in space: “My child can roll from back to tummy”. *Object manipulation* items assess the manipulation of a ball: “When I sit on the floor with my child and roll a ball towards him/her, he/she will roll the ball back to me”.

#### Social-emotional Scale

##### Details on questionnaires

In the following sections, we provide additional information of the established questionnaires, which are used to assess children’s social-emotional development in the APP.

##### Infant Behavior Questionnaire - Revised (IBQ-R)

The original IBQ was developed by Rothbart (1981) and revised as IBQ-R by Gartstein and Rothbart (2003). The full version of the IBQ-R includes 14 scales and 191 items. For feasibility and efficiency reasons, we implemented the Very Short Form version (Putnam et al., 2014, IBQ-R-VSF,) that includes 37 items in three broad scales (Positive Affectivity/Surgency, Negative Emotionality, Orienting/Regulatory Capacity). For the non-English versions, we used the available translations (German: (Kristen et al., 2007) and slightly revised by Vonderlin et al., (2012); Italian: (Montirosso et al., 2011); French: (Cascales, 2011)).

##### Early Childhood Behavior Questionnaire (ECBQ)

The ECBQ was developed by Putnam et al., (2006). The original questionnaire includes 18 scales and 201 items. We used the very short form version (Putnam et al., 2010) that includes 36 items in three scales (similar to the IBQ-R-VSF: Positive Affectivity/Surgency, Negative Emotionality, Orienting/Regulatory Capacity). For the non-English versions, we used the available translations (German: (Kirchhoff and Fuchs, n.d.); French: (Goupil, n.d.); Italian: (Cozzi et al., 2013)).

##### Children’s Behavior Questionnaire (CBQ)

The CBQ was developed by Rothbart et al., (2001). The original questionnaire includes 15 scales and 195 items. We used the very short form version (Putnam and Rothbart, 2006) that includes 36 items and three scales (similar as in the IBQ-R-VSF: Surgency, Negative Affect, and Effortful Control). For the non-English versions, we used the available translations (German: (Koglin & Petermann, 2007; Nikolaizig, 2007); French: (Lafortune et al., n.d.); Italian: (Matricardi, n.d.)).

##### Children’s Social Understanding Scale (CSUS)

Children’s ToM is usually measured via a range of laboratory paradigms that assess the understanding of mental states such as beliefs, desires, emotions, and intentions (e.g., Wellman, 2007). We implemented the CSUS (Tahiroglu et al., 2014), a caregiver-report ToM measure. It includes six scales (belief, knowledge, perception, desire, intention, emotion) with a total of 42 items which have been shown to be a measure of individual differences in children’s ToM with very high internal consistency, test–retest reliability, and predictive validity.

##### Answer options

The items used in the different scales describe behavior that frequently occur in everyday contexts (e.g., “When being dressed or undressed,”, “When playing outdoors”, “When told no”). Accordingly, caregivers are asked to indicate how often their child showed a particular behavior during the last week (i.e., the past 7 days) on a seven-point Likert-style format ranging from “never” to “always”. We applied the same scales as used in the original questionnaires.

## Footnotes

1 The respective answer options only allowed a systematic depiction of the AoAs for these two scales

2 Because there were only very few caregivers outside of this age window we categorized them as test users.

3 In the latest version of the APP, it is no longer possible to indicate an impossible AoA.

## Declarations

### Open Practices Statement

The items of all the scales and information about data protection are available on https://osf.io/ar7xp/. The data sets generated during and/or analyzed during the current study are not publicly available due to the current data protection guidelines, see section Data security and data protection but are available from the corresponding author on reasonable request.
